# Association Studies of MMP-9 in Parkinson’s Disease and Amyotrophic Lateral Sclerosis

**DOI:** 10.1371/journal.pone.0073777

**Published:** 2013-09-09

**Authors:** Xianghua He, Lifang Zhang, Xiaoli Yao, Jing Hu, Lihua Yu, Hua Jia, Ran An, Zhuolin Liu, Yanming Xu

**Affiliations:** 1 Department of Neurology, West China Hospital, Sichuan University, Chengdu, Sichuan Province, China; 2 Department of Neurology, General Hospital of Ning Xia Medical University, Yinchuan, Ningxia Province, China; 3 Department of Neurology, Third Hospital of Hebei Medical University, Sijiazhuang, Hebei Province, China; 4 Department of Neurology, First Affiliated Hospital, Sun Yat-sen University, Guangzhou, Guangdong Province, China; University Hospital La Paz, Spain

## Abstract

Parkinson’s disease (PD) and amyotrophic lateral sclerosis (ALS) share several clinical and neuropathologic features, and studies suggest that several gene mutations and polymorphisms are involved in both conditions. Matrix metalloproteinase-9 (MMP-9) is implicated in the pathogenesis of PD and ALS, and the C(−1562)T polymorphism in the *MMP-9* gene leads to higher promoter activity. We therefore investigated whether this polymorphism predisposes to both PD and sporadic ALS (sALS). Samples from 351 subjects with PD and 351 healthy controls from two major cities in China were compared, while samples from 226 subjects with sALS were compared to the same number of controls from three centers in China. A possible association between the C(−1562)T polymorphism in the *MMP-9* gene and PD or sALS was assessed by restriction fragment length polymorphism (RFLP) analysis. Our results show a significant association between the C(−1562)T polymorphism in the *MMP-9* gene and risk of PD (odds ratio = 2.268, 95% CI 1.506–3.416, p<0.001) as well as risk of sALS (odds ratio = 2.163, 95% CI 1.233–3.796, p = 0.006), supporting a role for *MMP-9* polymorphism in the risk for PD and sALS.

## Introduction

Parkinson’s disease (PD) and amyotrophic lateral sclerosis (ALS) are neurodegenerative disorders whose etiology and pathogenesis are poorly understood. Nevertheless, various biochemical, environmental and genetic mechanisms have been proposed for both conditions [1]–[3].

Interestingly, numerous studies have described individuals who demonstrate a neurodegenerative ‘overlap’ syndrome, comprising idiopathic parkinsonism, dementia, and ALS [4]–[8]. Epidemiological studies have shown that relatives of ALS patients are at increased risk of developing PD [9]–[10]. In addition, studies have demonstrated that mutations in TAR DNA-binding protein (TARDBP), variants of angiogenin (ANG), polymorphisms within axon guidance pathway genes, expanded ataxin 2 (ATXN2) repeats and hexanucleotide repeat expansions in C9ORF72 gene are involved in both PD and ALS [11]–[18].

Matrix metalloproteinases (MMPs) are proteases that remodel the extracellular matrix (ECM). Matrix metalloproteinase-9 (MMP-9), a major component of the basement membrane, may contribute to the pathogenesis of neurodegenerative diseases such as Alzheimer’s disease, PD and ALS by inducing neuronal death [19]–. Levels of tissue inhibitors of MMPs including MMP-9 are elevated in the cerebrospinal fluid of individuals with PD and in the skin, serum, and cerebrospinal fluid of individuals with ALS [20], [22]–[24].

These findings linking MMP-9 to PD and ALS suggest that polymorphisms in the *MMP-9* gene may affect susceptibility to the developing both conditions. Only few studies have examined this possibility, and the results have been inconsistent. The C(−1562)T polymorphism, in which the T allele shows higher promoter activity than the C allele [25], was found not to be associated with ALS in a Polish population [26], while in another small population from Poland, Iłżecka found elevated levels of an extracellular MMP inducer in the serum of patients with ALS, as well as an association between the levels of this inducer and the clinical severity of ALS [27]. At the same time, no data have been published on the possible association of the C(−1562)T polymorphism and PD.

Therefore, we investigated a series of Chinese patients with PD or sALS to determine whether the C(−1562)T polymorphism in the *MMP-9* gene predisposes to either or both conditions.

## Subjects and Methods

### 2.1 Subjects

In our case-control study, 351 Chinese patients with sporadic PD and 351 healthy, ethnically matched control subjects were consecutively recruited from two movement disorder centers: West China Hospital, Sichuan University, located in southwest China; and the First Affiliated Hospital, Sun Yat-sen University, located in southeast China. Clinical diagnosis of PD was established by two independent movement disorder specialists according to accepted criteria [28]. Patients with one or more relatives diagnosed with PD were excluded. We defined early-onset PD (EOPD) as showing an age at onset <50 years (n = 118), and the mean age of these patients was 42.5±5.8 years (range 25–49). The mean age at onset of patients with late-onset PD (LOPD; n = 233) was 60.8±6.8 years (range 50–78). The control sample for PD group was composed of unrelated healthy individuals matched by age and sex. The average age for PD patients is 54.5±11.1 years, and for controls is 53.2±10.9 years. There are no differences between PD patients and the controls in age and gender.

Patients with sALS were recruited from three medical centers: the Department of Neurology, Third Hospital of Hebei Medical University, Hebei Province, located in north China; the Department of Neurology, First Affiliated Hospital of Sun Yat-sen University, Guangdong Province, in southeast China; and the Department of Neurology, West China Hospital, Sichuan University, located in southwest China. All patients satisfied the 2000 El Escorial criteria for definite or probable ALS, and all patients and controls were ethnic Han Chinese. The control sample was composed of unrelated healthy individuals matched by age and sex. The average age for ALS patients is 52.0±11.5 years, and for controls is 51.0±12.6 years. There are no significant differences between ALS patients and the controls in age and gender.

Separate control groups were used for the PD and sALS patient groups because the average age and sex ratio of patients with PD were significantly different from those of patients with sALS. The protocol of this study was approved by the Ethics Committee of all participants: Sichuan University, Sun Yat-sen University and Hebei Medical University. All individuals gave informed consent in writing.

### 2.2 Genotyping

Genomic DNA was extracted from peripheral blood leukocytes using the standard method of proteinase K digestion, followed by phenol-chloroform extraction and ethanol precipitation. Samples were genotyped for the C(−1562)T polymorphism using polymerase chain reaction-restriction fragment length polymorphism (PCR-RFLP) analysis. Amplified DNA was sequenced using the following primers: 5′-TTATCTCCATCTCACAGTCTCATT-3′ (sense) and 5′-TATTTTTGGGGGGTGTAGTATC-3′ (antisense). PCR reactions (25 µl) contained 0.1 µg genomic DNA, 10 pmol of each primer, 10 pmol dNTP, and 1.5 units of Taq polymerase (TaKaRa, Japan), and standard PCR buffer. Amplification conditions were an initial denaturation at 94°C for 5 min, followed by 35 amplification cycles consisting of denaturation at 94°C for 30 s, annealing at 56°C for 30 s, and extension at 72°C for 30 s, with a final extension at 72°C for 5 min. All amplification reactions were resolved by 2% agarose gel electrophoresis to confirm the specific PCR products. These products were then digested overnight in a total volume of 10 µl at 37°C with the restriction endonuclease Sph I (New England Biolabs, USA), separated on a 3% agarose gel and stained with ethidium bromide.

### 2.3 Statistics

Chi-square (χ^2^) analysis was used to assess differences in allele and genotype frequencies between patient and control groups. A Hardy–Weinberg equilibrium (HWE) program was used to test whether alleles deviated from HWE. Odds ratios (ORs) and 95% confidence intervals (CIs) were calculated. A two-tailed P-value <0.05 was considered significant. Statistical analysis was performed using the Statistical Package for the Social Sciences (SPSS) for Windows (version 15.0; SPSS Inc., Chicago, IL).

## Results

We analyzed and compared the frequencies of genotypes and alleles of the C(−1562)T polymorphism in the *MMP-9* gene in 351 patients with PD and 351 control individuals, and in 226 sALS patients and 226 control individuals. Genotype distributions were in HWE in the PD group (p = 0.123) and the corresponding control group (p = 0.808), as well as in the ALS group (p = 0.192) and the corresponding control group (p = 0.464). Because of the relatively low frequency of the TT genotype, we combined the CT and TT genotypes into the CT+TT group, which we compared with the CC genotype group (Table 1). And CC vs (CT+TT) statistical test corresponds to a genotypic test under a dominant model. There were significant between-group differences in allele frequencies (for PD:*P*<0.001; for ALS: *P* = 0.002) and heterozygous/homozygous genotype (for PD: *P*<0.001; for ALS: *P* = 0.006) for the C(−1562)T polymorphism (Table 1).

**Table 1:** Genotype and allele frequencies of the C(−1562)T polymorphism of the *MMP-9* gene in patients with PD or sALS and healthy controls.

	n	Genotype, n(%)			Allele, n(%)	
		CC	CT	TT	C	T
PD:						
Patients	351	270 (76.9)	72 (20.5)	9 (2.6)	612 (87.2)	90 (12.8)
Controls	351	310 (88.3)	40 (11.4)	1 (0.3)	660 (94.0)	42 (6.0)
ALS:						
Patients	226	185 (81.8)	37 (16.4)	4 (1.8)	407 (90.0)	45 (10.0)
Controls	226	205 (90.7)	21 (9.3)	0	431 (95.4)	21 (4.6)

PD: CC vs CT/TT: OR 2.268, 95% CI 1.506–3.416, p<0.001.

C allele vs T allele: OR 2.311, 95% CI 1.577–3.387, p<0.001.

ALS: CC vs CT/TT: OR 2.163, 95% CI 1.233–3.796, p = 0.006.

C allele vs T allele: OR 2.269, 95% CI 1.328–3.876, p = 0.002.

We then analyzed whether heterozygous and homozygous C(−1562)T carriers with PD or sALS differed in their clinical characteristics. Heterozygous and homozygous C(−1562)T carriers and non-carriers of C(−1562)T polymorphism with PD did not differ significantly in gender ratio, age at onset, onset symptoms, Hoehn-Yahr stage or United Parkinson’s Disease Rating Scale (UPDRS) (Table 2). Similarly, carriers and non-carriers with ALS did not show significant differences in gender, age at onset, or onset symptoms (Table 2). Furthermore, we found that there were more female cases positive (heterozygous CT and/or homozygous TT) than male (27.8% vs. 19.2%) in PD group and more male cases positive (heterozygous CT and/or homozygous TT) than female (20.4% vs. 12.5%) in ALS group, whereas no similar phenomenon was found in PD controls or ALS controls. (Table 2).

**Table 2:** Clinical characteristics of patients and controls with PD or sALS, according to genotype at the C(−1562)T polymorphism of the *MMP-9* gene.

Characteristic		C(−1562)T genotype		
	n	CC	CT/TT	p value
**PD:**				
Gender				
male, n(%)	193	156(80.8)	35/2(19.2)	
female, n(%)	158	114(72.2)	38/6(27.8)	0.074
Age at onset (years)				
Total cohort		54.889±10.286	53.741±12.453	0.403
EOPD		42.886±5.116	41.267±7.325	0.185
LOPD		60.692±6.385	61.078±8.289	0.722
Onset symptoms^a^, n(%)				
Resting tremor	185	138(51.1)	47(58.0)	
Bradykinesia-rigidity	121	99(36.7)	22(27.2)	
Mixed symptoms	41	31(11.5)	10(12.3)	
Others	4	2(0.7)	2(2.5)	0.052^a^
Hoehn-Yahr stage		2.252±0.830	2.327±0.779	0.468
**Controls for PD**				
Gender				
Male	203	178 (87.7)	25/0(12.3)	
Female	148	132(89.2)	15/1(10.8)	0.438
Age		53.174±10.874	53.024±11.239	0.908
**ALS**				
Gender				
Male	162	129(79.6)	29/4(20.4)	
Female	64	56(87.5)	8/0(12.5)	0.280
Age at onset (years)		54.648±10.889	54.228±10.903	0.108
Onset symptoms				
Limb onset	198	163(82.3)	35(17.7)	
Bulbar onset	28	21(75.0)	7(25.0)	0.351
**Controls for ALS**				
Gender				
Male	157	143 (91.1)	14/0(8.9)	
Female	69	62 (89.9)	7/0(10.1)	0.805
Age		51.005±12.499	51.429±13.912	0.639

a Multiple-factor comparison of onset symptoms showed no significant difference (p = 0.052).

CC vs (CT+TT) statistical test corresponds to a genotypic test under a dominant model.

## Discussion

To the best of our knowledge, this is the first report investigating the association between the C(−1562)T polymorphism in the *MMP-9* gene and risk of PD, and the first study to confirm a positive association between the C(−1562)T polymorphism and risk of sALS. In our hospital-based case-control study, we demonstrated that the C(−1562)T polymorphism is a potential risk factor for both PD and sALS in the Han Chinese population of mainland China. In fact, we found that the polymorphism increases the risk of PD by approximately 2.3-fold and of sALS by nearly 2.2-fold.

These results contradict earlier observations in a Polish population involving 228 sALS patients [26]: in which 53 patients (23.2%) were identified as heterozygous and 7 (3.1%) as homozygous for the C(−1562)T polymorphism, while in control groups, 118 patients (27.6%) were identified as heterozygous and 6 (1.4%) as homozygous, and thus no significant association was observed between the C(−1562)T polymorphism of *MMP*-*9* gene and risk of sALS (p = 0.47). This discrepancy may be due to small population, to ethnic differences, and/or to genetic background, and should be confirmed in cohorts in other countries and in other ethnicities from Asian countries.

In our study, although our results show no significant differences by statistics in gender in both PD group and ALS group, they indicate that there is a trend that more female cases carrying heterozygous CT and/or homozygous TT in PD group and more male cases carrying heterozygous CT and/or homozygous TT) in ALS group. This is an important found which would be helpful for further study. We speculate that the C(−1562)T polymorphism may have different roles in the Pathological mechanism of PD and ALS, though it is associated with the risk of both PD and ALS. Further studies based on large samples and multi-centers are needed to confirm these new discoveries.

Although our results indicate an association between carriers and non-carriers of the C(−1562)T polymorphism and risk of PD and sALS, we did not find an association between C(−1562)T genotype and onset age, onset symptoms or severity of PD according to the Hoehn-Yahr stage and UPDRS. Similarly, we did not find an association between C(−1562)T genotype and onset age or onset symptoms of sALS.

Our finding that polymorphism in a single gene predisposes to two neurodegenerative disorders adds to the number of cases in which genetic mutations have been linked to neurodegenerative disease. For instance, mutations in the dynactin1 gene (*DCT1),* which is involved in axon maintenance, have been strongly linked to Perry syndrome, which is a parkinsonian disorder with TDP-43-positive pathology; frontotemporal lobar degeneration (FTLD); and ALS [29]–[30]. Furthermore, Researchers found that expansions of hexanucleotide repeat in C9ORF72 gene are detected in 9.3% patients with ALS, in 5.2% patients with FTLD, and in 0.7% patients with PD [15]. Recently, a large collaborative study involving patients at 15 medical centers showed that the angiogenin gene *ANG* is a link between PD and ALS [12]. Interestingly, it has recently been suggested that mutations in TARDBP, polymorphisms in axon guidance pathway genes, and expanded ATXN2 repeats are all associated with both PD and ALS [11], [13]–[14].

Several mechanisms may explain how MMP-9 contributes to ALS. MMPs can be induced by inflammatory cytokines like IL-1 and TNF-α, which are among the most potent transcriptional activators of MMP-9 [31]. In ALS, microglial activation with increased levels of IL-1 and TNF-α is a well known phenomenon that could contribute to elevated MMP-9 concentrations [32]. Overexpression of MMP-9 and its release at the synapse may destroy the structural integrity of the surrounding ECM, thereby contributing to the pathogenesis of ALS [33]. MMP activity and gene expression were shown to be influenced by reactive oxygen species (ROS) implicated in oxidative stress, which is supposed to be a key pathomechanism in ALS [34]. However, later study did not show direct link of MMP concentrations to oxidative stress [13]. Moreover, MMP-9 can cleave substance P, which acts as a neurotransmitter in the spinal cord and plays a role in the physiology of healthy individuals and in the pathophysiology of ALS [33]. In post-mortem ALS brain tissue, skin, plasma and in cerebrospinal fluid, increased MMP-9 and matrix metalloproteinases (TIMPs) were reported [20], [22]–[24]. MMP-9 in the transgenic mouse model of ALS is supposed to contribute to neuron death because of neurotoxicity [35]. Another study with transgenic ALS mice model suggests that pharmacologic inhibition of MMPs with a synthetic inhibitor in the early stages of disease prolongs survival, suggesting a role for MMPs in early pathogenesis [36]. Thus, it is reasonable to speculate that the C(−1562)T polymorphism of the *MMP*-*9* gene affects MMP-9 activity and thereby contributes to ALS pathophysiology.

MMPs have also been proposed to cleave intracellular substrates, and such intracellular proteolysis has been linked to PD [37]. In addition, the overexpression of alpha-synuclein characteristic of PD may stimulate MMP-9 activity: overexpression of alpha-synuclein in rat primary astrocytes increased MMP-9 activity by stimulating microglia to activate PAR-1 and amplify microglial inflammatory signals [38]–[39]. Treatment with MMP-9 inhibitors attenuated the neuronal cell death induced by the dopaminergic neurotoxins 6-OHDA and MPP(+) [39], and mechanistic studies showed that both 6-OHDA and MPP(+) can induce *MMP*-*9* promoter activity, suggesting that controlling MMP-9 expression may have therapeutic potential in PD [21].

In addition to the association with ALS and PD, MMP-9 has also been linked to Alzheimer’s disease (AD) and Huntington’s disease (HD) [20]. These findings may suggest that MMP-9 is involved in neurodegenerative disorders per se, or that these seemingly different diseases are part of the same neurodegenerative spectrum.

Our study has shown, for the first time, that the C(−1562)T polymorphism is likely to constitute a risk factor for both PD and sALS in China. Although the sample size was moderate, which limited the statistical power of our study, the fact that subjects were recruited from distant regions of the country should improve our genetic coverage. Nevertheless, our results should be independently confirmed in larger-scale studies conducted in China and in other countries. Also, more studies should test samples on ANG gene, and the trinucleotide expansions ATXN2 gene in PD and ALS patients. In particular, future studies should ensure more adequate representation of women in the sample. Such studies should help elucidate the association of this polymorphism with the pathogenesis of PD and ALS.
